# A bright light on myosin to study skeletal muscle relaxation

**DOI:** 10.1113/JP280664

**Published:** 2020-10-01

**Authors:** Ricardo A. Galli, Coen A. C. Ottenheijm

**Affiliations:** ^1^ Department of Physiology Amsterdam UMC (location VUmc) Amsterdam The Netherlands

**Keywords:** muscle kinetics, thick filament, X‐ray diffraction

Muscle contraction is the product of highly coordinated intricate interplay between the actin‐based thin filament and the myosin‐based thick filament, both key components of the sarcomere, the smallest contractile unit in muscle. During muscle activation, the number of force‐generating myosin molecules bound to actin rapidly builds and high forces are generated; during relaxation, this number rapidly decreases, bringing force down and allowing lengthening of passive muscle to occur.

At the level of the myofilament proteins, several conformational changes occur during muscle activation and relaxation. These changes have been extensively studied during muscle activation, using a range of methodologies including electron microscopy, low‐angle X‐ray diffraction, nuclear magnetic resonance and molecular dynamics simulations. In brief, during activation Ca^2+^ binds to troponin C, which then undergoes a conformation change promoting the opening of a hydrophobic pocket. This opening accommodates the binding of the troponin I switch peptide, causing downstream changes in the position of tropomyosin. This exposes myosin binding sites on the actin filament, so that myosin heads can bind and subsequently pull on actin to generate force. Sarcomere activation is not only regulated at the thin filament. The thick filament also has structural ‘OFF’ and ‘ON’ states, which are controlled in part by the external load on the muscle, so that the myosin heads only attach to actin and hydrolyse ATP when they are required.

Whereas the kinetics of muscle activation and its associated structural changes in myofilament proteins have been widely studied (reviewed in Malcolm Irving, 2017), the changes during muscle relaxation are much less well understood, despite its crucial importance for muscle contractility and disease (e.g. De Winter *et al*. 2020). Muscle relaxation is associated with a decrease in cytosolic Ca^2+^ ions, as they are sequestered in the sarcoplasmic reticulum. Interestingly, cytosolic [Ca^2+^] decreases relatively quickly whereas the time course of mechanical relaxation is longer: while force is decreasing at a constant and relatively slow rate, the cytosolic [Ca^2+^] is already at a low, constant level. During this phase of relaxation, the lengths of sarcomeres along the muscle fibre remain close to the lengths during tetanus activation (i.e. isometric relaxation). During the final phase of relaxation, sarcomere lengths become heterogeneous (i.e. chaotic relaxation). Previous work showed that during the isometric relaxation phase, myosin heads progressively detach from actin, but they remain in a disordered detached state and do not adopt the resting configuration around the thick filament backbone (Brunello *et al*. 2009). Thus, during this phase, the thick filament backbone remains in the activated ‘ON’ state. During the chaotic relaxation phase, the thick filament backbone transitions to the ‘OFF’ state and myosin heads move back to the thick filament backbone. These data were obtained with experiments in frog muscle fibres at 4°C. Although these data provided valuable insights it was unclear whether they translate to mammalian muscle, which slightly differs in structure and in the temperature it operates at. In the elegant study by Ma *et al*. (2020), published in the current issue of the *Journal of Physiology*, myosin‐based conformational changes during skeletal muscle relaxation were investigated in mice, at near physiological temperatures. At the BioCAT beamline at the Advanced Photon Source, Argonne National Laboratory, soleus muscle was dissected from mice and mounted between a force transducer and a length motor. Muscles were electrically stimulated at 22°C and simultaneously exposed to the X‐ray beam. This approach allowed for the determination of time‐resolved conformational changes in myosin during muscle relaxation. Overall, the data presented by Ma *et al*. are in line with the previous findings in frog muscle and indicate that during the first phase of muscle relaxation myosin heads detach from actin but remain in the proximity of actin. An interesting finding in the study by Ma *et al*. was that if muscles overexpress the naturally occurring myosin activator 2′‐deoxy‐ATP (dATP), with dATP levels at only 1% of the total ATP pool, the detachment rate of myosin heads is initially slower and they remain in an ‘ON’ state near the thin filaments. This might prime the myosin heads, facilitating subsequent contractions. This ‘priming’ of the myosin heads was supported by molecular dynamics simulations, which suggested that dATP induces conformational changes in the myosin heads that promote interaction with actin. After this initial, slow response the myosin heads regain their ordered relaxed configuration more rapidly. This dual effect of dATP could only be identified due to the high time resolution of the X‐ray studies. X‐ray studies in striated muscle are not novel (they were pioneered in the 1960s (Elliott *et al*. 1965; Huxley *et al*. 1965) and are technically demanding, but to date they are the most powerful approach for the simultaneous assessment of force kinetics and Ågstrom‐scale conformational changes in myofilament proteins.

In addition to their contribution to basic muscle function and structure, the findings by Ma *et al*. might provide insights into muscle disorders associated with abnormal kinetics of relaxation, such as Brody myopathy (caused by mutations in *ATP2A1*), nemaline myopathy type 6 (caused by mutations in *KBTBD13*), and distal arthrogryposis (mutations in *MYBPC1*). There is a lack of drugs that target abnormal relaxation kinetics. Thus, improving the understanding of the mechanisms that control sarcomere relaxation is of utmost importance, as they might identify targets for drug development. The data by Ma *et al*. indicate that the effects of dATP on the kinetics of muscle relaxation are significant, albeit subtle, at least in the soleus muscle of mice. Importantly, their data showcase that the combination of low‐angle X‐ray diffraction with simultaneous force measurements in intact mammalian muscle should be a central approach for future studies that aim to identify compounds for the treatment of neuromuscular disorders associated with impaired muscle relaxation kinetics. Combining this approach with molecular dynamics simulations will provide a highly complementary look at myofilament structure and the effects of compounds thereon.

## Additional information

### Competing interests

None.

### Author contributions

RAG and CACO wrote the article.

### Funding

None.
